# Dysfunction of Collagen Synthesis and Secretion in Chondrocytes Induced by *Wisp3* Mutation

**DOI:** 10.1155/2013/679763

**Published:** 2013-03-19

**Authors:** Min Wang, Xiao-Fei Man, Ya-Qing Liu, Er-Yuan Liao, Zhi-Feng Shen, Xiang-Hang Luo, Li-Juan Guo, Xian-Ping Wu, Hou-De Zhou

**Affiliations:** ^1^Institute of Endocrinology and Metabolism, The Second Xiang-Ya Hospital of Central South University, Changsha, Hunan 410011, China; ^2^Department of Endocrinology and Metabolism, Xiang-Ya Hospital of Central South University, Changsha, Hunan 410008, China

## Abstract

*Wisp3* gene mutation was shown to cause spondyloepiphyseal dysplasia tarda with progressive arthropathy (SRDT-PA), but the underlying mechanism is not clear. To clarify this mechanism, we constructed the wild and mutated *Wisp3* expression vectors and transfected into human chondrocytes lines C-20/A4; *Wisp3* proteins subcellular localization, cell proliferation, cell apoptosis, and *Wisp3*-mediated gene expression were determined, and dynamic secretion of collagen in transfected chondrocytes was analyzed by ^14^C-proline incorporation experiment. Mutated *Wisp3* protein increased proliferation activity, decreased apoptosis of C-20/A4 cells, and aggregated abnormally in cytoplasm. Expression of collagen II was also downregulated in C-20/A4 cells transfected with mutated *Wisp3*. Wild type *Wisp3* transfection increased intracellular collagen content and extracellular collagen secretion, but the mutated *Wisp3* lost this function, and the peak phase of collagen secretion was delayed in mutated *Wisp3* transfected cells. Thus abnormal protein distribution, cell proliferation, collagen synthesis, and secretion in *Wisp3* mutated chondrocytes might contribute to the pathogenesis of SEDT-PA.

## 1. Introduction

Wnt-1-induced secreted protein 3 (*Wisp3*/CCN6) is a cysteine-rich protein that belongs to the cysteine-rich 61-connective tissue growth factor, nephroblastoma overexpressed CCN family members, maps to chromosome 6q21-22, and encodes a 354 amino acid secreted protein [1]. *Wisp3* proteins are characterized by an N-terminal secretory signal followed by four structural domains with partial sequence identity to insulin-like growth factor binding protein (IGFBP) (GCGCCXXC); Von Willebrand factor type C like motif, thrombospondin type 1 module, and a C-terminal cysteine knot-like domain (CK) putatively involved in dimerization [1, 2], and IGFBP can be upregulated by implementation of exercise [3]. The members of CCN family are multifunctional in which they are involved in regulation of cell adhesion, migration, proliferation, growth arrest, survival, apoptosis, differentiation, endochondral ossification, and extracellular matrix production [4–6].

*Wisp3* mutations have been demonstrated in most patients of an autosomal recessive hereditary cartilage metabolic disorder, spondyloepiphyseal dysplasia tarda with progressive arthropathy (SEDT-PA), or progressive pseudorheumatoid dysplasia (PPD), which characterized by deformation and limitation of most large and small joints clinically, and continuous degeneration and loss of articular cartilage pathologically [7–11]. In our previous work, we found a novel compound mutation (840delT/T1000C) of *Wisp3* in Chinese PPD kindred [12, 13]; the two probands carried a substitution mutation (1000T→C, Ser334Pro) in paternal allele, and a deletion (840delT) mutation in maternal allele that caused a truncated *Wisp3* protein to miss 43 residues in C-terminus [14], and we also discovered the biological behavior changes of the articular chondrocytes (ACs) separated from the patients [15]. *Wisp3* also had growth-, invasion-, and angiogenesis-inhibitory functions in inflammatory breast cancer (IBC) in vitro and in vivo [16] and was a key genetic determinant of the IBC phenotype [17]. However, the precise action of *Wisp3* in cartilage maintenance and metabolism and the mechanisms of SEDT-PA/PPD caused by *Wisp3* mutations have not been elucidated.

The present study was undertaken to investigate the subcellular localization and function of mutant *Wisp3* in chondrocytes. The results suggest that mutated *Wisp3* protein aggregated abnormally in cytoplasm, and mutated *Wisp3* failed to inhibit cell proliferation and modulate the expression of type II collagen in chondrocytes, which may be an important molecule mechanism involved in the pathogenesis of SEDT-PA/PPD.

## 2. Materials and Methods

### 2.1. Cell Cultures

The immortalized human chondrocytes cell lines C-20/A4 were derived from human juvenile costal cartilage and generated by infection with a replication defective retroviral vector expressing SV40 large T antigen. Cultures of C-20/A4 cells were maintained in DMEM/Ham's F-12(1 : 1,v/v) supplemented with 10% fetal bovine serum (FBS, Invitrogen, Carlsbad, USA) in a 5% CO_2_ incubator at 37°C and passaged at subconfluence every 5-6 days.

### 2.2. Wild Type and Mutant **Wisp3 ** Expression Construct

Human *Wisp3* was cloned from ACs cDNA by PCR amplification with *Wisp3*-specific primers, bearing Hind III and BamH I restriction enzyme sites at their flanking ends, for the purpose of subcloning into the expression vector pcDNA3.1(+) and pEGFP-C2 (Invitrogen). The primers used for cloning *Wisp3* into pcDNA3.1 were 5′-GTAAGCTTAGCGACATGCAGGGGCTCCTCTT-3′ (forward) and 5′-GCGGATCCTTACAGAATCTTGAGCTCAG-3′ (reverse), and pEGFP-C2 were 5′-TCAAGCTTCGACGTACAGGGGCTCCTCTT-3′ (forward, exclude start coden) and 5′-GCGGATCCTTACAGAATCTTGAGCTCAG-3′ (reverse). The amplified *Wisp3* gene products (~1.1 kb) were ligated to pGEM-T easy (Promega, Madison, WI, USA), and the products were used as templates in the PCR reaction of the site-directed mutagenesis (SDM). 

The mutants *Wisp3* (MUT^1000T/C^ and MUT^840delT^) were constructed using SDM separately. The mutant primers for MUT^1000T/C^ had the sequences 5′-CCTTGTGTGTGTCAGAGAAA-3′ (forward) and 5′-CCTTCTACGACACCTAATGT-3′ (reverse), and for MUT^840delT^ had the sequences 5′-AATTGTCTTTTCTGGATGCTCA-3′ (forward) and 5′-AAGGTTGAGAGGTTTCGACTTT-3′ (reverse). After confirmed by restriction endonuclease analysis and sequencing, the target fragments (WT-*Wisp3*, MUT^1000T/C^, and MUT^840delT^) were subcloned to Hind III and BamH I sites of expression vector pcDNA3.1(+) and pEGFP-C2. The recombined expression plasmids with pcDNA3.1(+) were used for all functional studies of wild and mutant *Wisp3*, and those with pEGFP-C2 were used for subcellular localization of wild and mutant *Wisp3* proteins.

### 2.3. Cell Transfection

Lipofectamine was used for transfecting C-20/A4 chondrocytes cell lines with the recombined plasmids and empty vector constructs. Briefly, cells (2.5~5 × 10^5^/mL) were plated 1 day before transfection in 6-well tissue culture plates (2 mL/well) and incubated at 37°C in 5% CO_2_. A complex of the plasmid DNA (<1 *μ*g) with 6 *μ*L PLUS reagent in 100 *μ*L of serum-free, antibiotic-free medium was prepared in a sterile microfuge tube for 15 minutes; dilute 4 *μ*L of Lipofectamine into 100 *μ*L of serum-free medium and added to each reaction mixture, and incubated at room temperature for additional 15–30 min. A similar complex was prepared for each well of a 6-well plate. The cells in each well of the plate were washed with sterile PBS and then added 800 *μ*L serum-free medium and the transfection mixture drop wise to each plate, and incubated for 4 h, after which 1 mL of culture medium with 5% FBS was added to each well. After 24 hours, the transfection mixture was replaced with fresh culture medium containing 10% FBS. The incubation was continued for an additional 24–26 hours, and the cells were used for observation by laser scanning confocal microscopy (LSCM) and harvested for either RNA or protein extraction. After 24 hours of transfection, the cells were placed in 25 cm^2^ flasks for stable transfection with selection by G418 (400 *μ*L /mL).

### 2.4. Wild and Mutant **Wisp3 ** Proteins Subcellular Localization

After 48 h of transiently transfection with WT-*Wisp3*/pEGFP-C2, MUT^1000T/C^/pEGFP-C2, MUT^840delT^/pEGFP-C2, or empty vector, cells were rinsed once with PBS after removal of culture medium, and then fixed for 15 min with freshly prepared 4% paraformaldehyde, and then incubated with 0.25% Triton X100 at 37°C for 20 min. 4′-6-Diamino-2-phenylindole-2HCl (DAPI; Sigma) was used at a final concentration of 100 ng/mL to stain cell nuclei. After washing three times with PBS at room temperature for 10 min, the fluorescence was observed under LSCM. 

### 2.5. Cell Viability Assay

Cell viability was determined using a standard 3-(4,5-dimethyl-2-thiazolyl)-2,5-diphenyl-2H-tetrazolium bromide (MTT) assay. Briefly, cells stably transfected with MUT^1000T/C^/pcDNA3.1(+), MUT^840delT^/pcDNA3.1(+), or empty vector were seeded 10^4^ cells/well into 96-well plates. Following 24 h in culture, 5 *μ*L MTT was added into each well and cells were incubated for an additional 3 h. The medium was discarded and the cells were solubilized in 100 *μ*L dimethyl sulphoxide (DMSO), and then shaken for 1 min, and incubated for 5 min at room temperature, and the absorbance at 570 nm was read on Micro ELISA reader (Molecular Devices, CA, USA).

### 2.6. Cell Cycle and Apoptosis Analysis

Cells stably transfected with MUT^1000T/C^/pcDNA3.1(+), MUT^840delT^/pcDNA3.1(+), or empty vector were seeded into in 25 cm^2^ flasks at a density of 2 × 10^5^ cells/mL and cultured for 24 h. After cultured in serum-free medium for 24 h, cells were digested with 0.05% trypsin-EDTA, rinsed with PBS, fixed with 75% ethanol over night at 4°C, and stained with propidium iodide. Cell cycle and apoptosis were evaluated using FAC flow cytometry (BD Biosciences). Cell proliferation index (PI) was calculated using the equation (PI) = (G2+S)/(G1+S+G2).

Apoptosis was also studied morphologically using fluorescent dyes that intercalate DNA. Acridine orange stains DNA bright green, allowing visualization of the nuclear chromatin pattern. Apoptotic cells have condensed chromatin that is uniformly stained. Ethidium bromide stains DNA orange, but is excluded by viable cells. Cells stably transfected with MUT^1000T/C^/pcDNA3.1(+), MUT^840delT^/pcDNA3.1(+), or empty vector were stained by acridine orange and ethidium bromide, respectively, and observed using LSCM.

### 2.7. RNA Extraction and RT-PCR

The C-20/A4 chondrocytes were transfected with WT-*Wisp3*, MUT^1000T/C^, MUT^840delT^, or empty vector, and the total RNA was extracted using TRizol Reagent (Invitrogen). RNA was treated with RNase-free DNase (Promega). Reverse transcriptase-PCR (RT-PCR) was performed using a reverse transcription kit according to the instructions of the manufacturer (Invitrogen). Primers specific for type II collagen, type I collagen, *SOX9*, fibronectin, *MMP-1*, and *β*-actin were used for estimating the levels of expression of the corresponding mRNA. During cDNA synthesis, 2 *μ*g of RNA was used for each specimen, and 30 cycles of PCR were carried out. The *β*-actin gene was used as an internal control.  Table 1 summarizes the primer pairs and experimental conditions used for RT-PCR analysis.

### 2.8. Preparation of Whole Cell Protein Lysates and Western Blot

To prepare whole cell lysates, cells transfected with WT-*Wisp3*, MUT^1000T/C^, MUT^840delT^, or empty vector were rinsed once with precooling PBS after removal of culture medium and treated with precooling Triton lyses buffer (50 mmol/L Tris-HCL, PH8.0 containing 150 mmol/L NaCL, 1%Triton X100, 10 mmol/L EDTA, 0.2% NaN_3_, 10 *μ*g/mL Aprotinin, and 1 *μ*g/mL phenylmethylsulfonyl fluoride) on ice for 20 minutes and the protein concentrations were determined using Bradford protein assay. Approximately 50 *μ*g of each cell lysate was mixed with 2×SDS gel-loading buffer (100 mmol/L Tris-HCL, PH6.8, and 200 mmol/L DDT, 4% SDS, 0.2% bromphenol blue, and 20% glycerol) and then heated to 95°C for 5 min. The samples were loaded onto a polyacrylamide gel (7.5 for type II collagen, 12% for *Wisp3* and *β*-actin), and prestained molecular weight standard (Bio-Rad, CA, USA) was also loaded onto the gels. After electrophoresis, the SDS-PAGE separated proteins were transferred to a nitrocellulose (GE, Pittsburgh, USA). The membrane was blocked with 5% nonfat milk in PBS for 60 min and then incubated with 2 *μ*g/mL goat monoclonal antihuman type II collagen, *Wisp3*, or *β*-actin (Santa Cruz, CA, USA) for 60 min. After extensive washing with PBS, the membrane was reprobed with mouse anti-goat IgG conjugated with horseradish peroxidase (GE) at 1 : 1000 in PBS for 1 h at room temperature. Blots were visualized with chemiluminescence as described previously [18].

### 2.9. ^14^C-Proline Incorporation Analysis

Chondrocytes were seeded into 24-well plates (5 × 10^4^ cells/cm^2^) in DMEM with 10% FBS for 24 hours, then cultured in 500 *μ*L of serum-free DMEM for 4 hours. For ^14^C-proline incorporation, each well was added with 10 *μ*Ci of ^14^C-proline (100 *μ*Ci/mL) (GE) and 100 *μ*g/mL aminopropionitrile, then incubated for 2 h; cells were rinsed five times with PBS, and complete medium was added and incubated at 37°C for 0, 30, 60, 120, 180, 240, and 300 minutes; supernatant and cell lysates were collected at these time points. For the collection of cell lysates, cells were rinsed two times with PBS after the supernatant collection, two times with 5% cold trichloroacetic acid, two times with 80% ethanol and finally lysed at 37°C for 2 h in 0.5 mL of 10 mM EDTA; 0.2 mL aliquots of the lysates and supernatant were dissolved in 10 mL Ecoscint H (Prolabo, Briare, France) separately and counted by scintillation; the quantification of intracellular collagen content and extracellular secretion was determined by the radioactivity in the cell lysate and supernatant, and the ratio of extracellular collagen secretion to intracellular collagen content was counted.

### 2.10. Statistical Analysis

SPSS 11.0 software was used for statistical analysis and data are presented as the mean ± SD, with the exception of gene analysis data. Data were compared using one-way ANOVA or the student's *t*-test. All experiments were repeated at least 3 times; the representative experiments are shown.

## 3. Results

### 3.1. Abnormal Protein Localization of Cells Transfected with **Wisp3 ** Mutants

**Figure 1:**
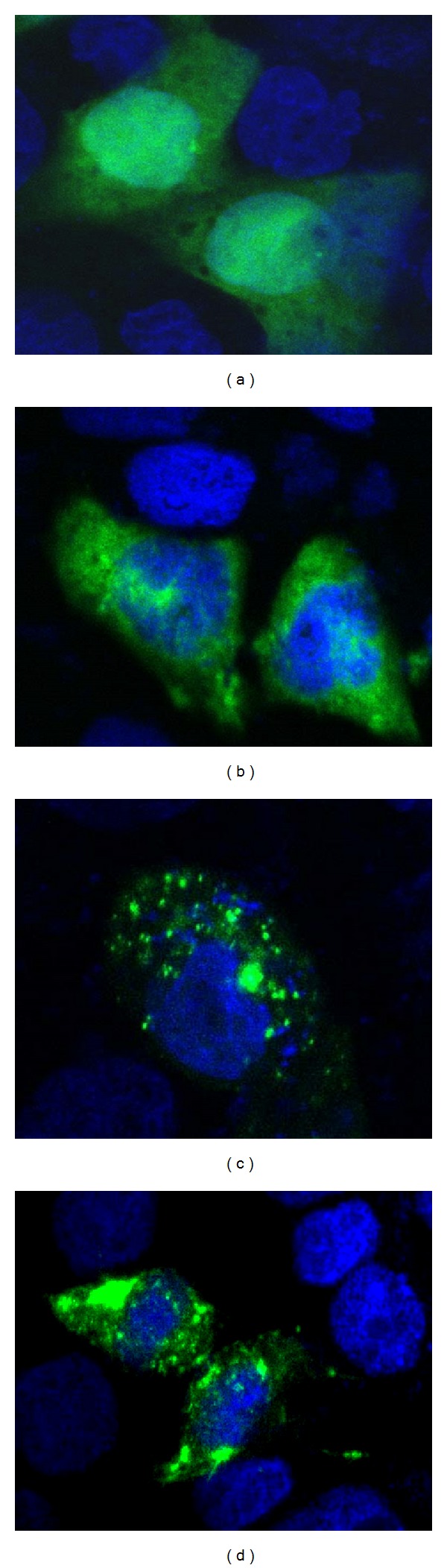
Localization of wild and mutated *Wisp3* protein in C20/A4 cells by confocal microscope. Recombined plasmids WT-*Wisp3*/pEGFP-C2, MUT^1000T/C^/pEGFP-C2, and MUT^840delT^/pEGFP-C2 were transfected transiently into human chondrocyte cell line C20/A4, and pEGFP-C2 vector was used as a control. The cells were observed using a confocal laser scanning microscope after 48 hours of transfection at magnification 1000x. (a) EGFP; (b) WT-*Wisp3*; (c) MUT^1000T/C^; (d) MUT^840delT^; Green fluorescence indicate the *Wisp3* EGFP fusion protein. Blue fluorescence shows cell nuclei dye by DAPI. Note the distribution of WT-*Wisp3* in cytoplasm and cell membrane uniformly. In contrast, the majority of MUT^1000T/C^ and MUT^840delT^ were aggregated to speckles or agglomerates in cytoplasm.

The recombined plasmids WT-*Wisp3*/pEGFP-C2, MUT^1000T/C^/pEGFP-C2, and MUT^840delT^/pEGFP-C2 were transfected transiently into human chondrocytes cell line C-20/A4, and pEGFP-C2 vector was used as a control. The expression and localization of green fluorescence protein (GFP) fusion proteins were observed using LSCM after 48 hours of transfection. GFP signal was distributed throughout the cells transfected with pEGFP-C2 vector (Figure 1(a)) and uniformly in cytoplasm and cell membrane transfected with WT-*Wisp3*/pEGFP-C2 (Figure 1(b)); however, the fluorescence signal aggregated to speckles or agglomerates in cytoplasm transfected with MUT^1000T/C^/pEGFP-C2 and MUT^840delT^/pEGFP-C2 (Figures 1(c) and 1(d)).

### 3.2. Increased Cell Proliferation and Decreased Cell Apoptosis Ratio of Cells Transfected with **Wisp3 ** Mutants

**Figure 2:**
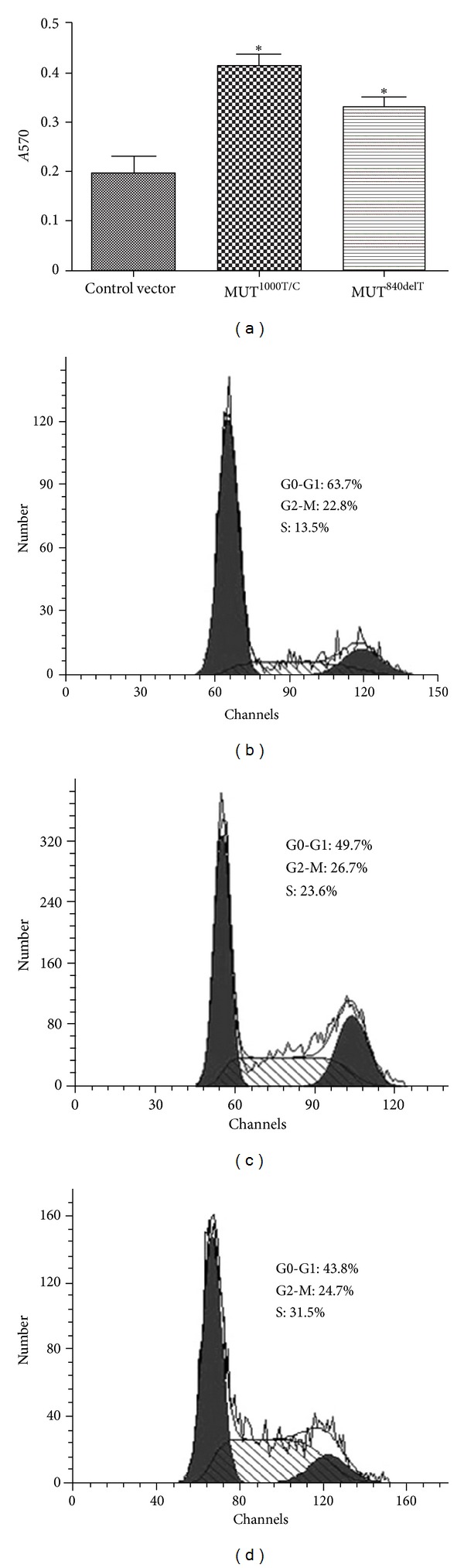
Cell viability and cycle analysis in C20/A4 cells transfected with *Wisp3*. Cells stably transfected with MUT^1000T/C^/pcDNA3.1(+), MUT^840delT^/pcDNA3.1(+) or empty vector, and cell viability (a) were determined by MTT and cell cycle was evaluated using flow cytometry. (b) Empty vector; (c) MUT^1000T/C^; (d) MUT^840delT^. *P < 0.01 compared with C20/A4 cells transfected with empty vector.

MTT assays showed cell viability in C-20/A4 cells stably transfected with MUT^1000T/C^/pcDNA3.1(+) and MUT^840delT^/pcDNA3.1(+) was obviously higher (0.38 ± 0.03 and 0.42 ± 0.04, *P* < 0.01) than that in cells stably transfected with pcDNA3.1(+) (0.24 ± 0.02) (Figure 2(a)).

Overproliferation of cells stably transfected with mutant *wisp3* was further demonstrated by flow cytometry analysis, which indicated that the cell numbers in the G2-M plus S phases were significantly higher than that of control cells (33.6 ± 4.0%, *P* < 0.05, Figure 2(b)), with a proliferation index of 49.8 ± 5.0% and 53.2 ± 4.5%, respectively (Figures 2(c) and 2(d)).

**Figure 3:**
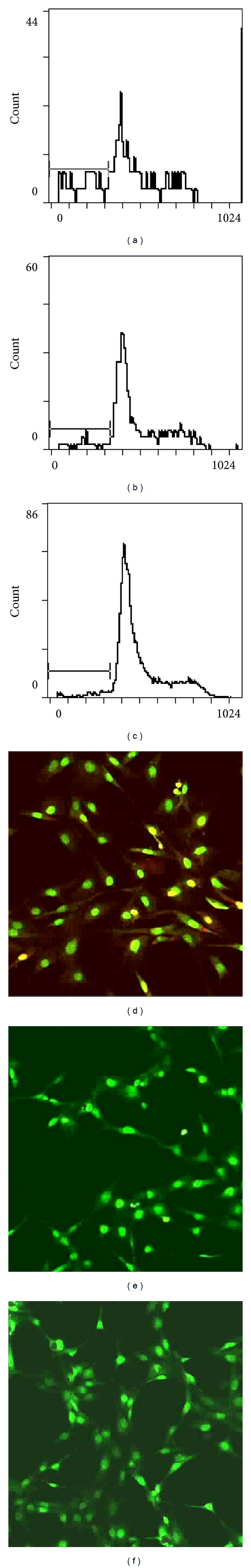
Cell apoptosis analysis in C20/A4 cells transfected with mutant *Wisp3* Cells stably transfected with MUT^1000T/C^/pcDNA3.1(+), MUT^840delT^/pcDNA3.1(+), or empty vector; cell apoptosis was evaluated by using FAC flow cytometry (a–c) and acridine orange/ethidium bromide staining (d–f) (apoptotic cells stained with yellow, condensed, or fragmented nuclei) analysis. (a) and (d) empty vector apoptosis rate is 27.1%; (b and e) MUT^1000T/C^ apoptosis rate is 9.2%; (c and f) MUT^840delT^ apoptosis rate is 7.8%. Magnification is 200 x.

The apoptosis rate of control cells was 26.1 ± 4.0% after cultured in serum-free medium for 24 h (Figure 3(a)), while that of cells stably transfected with MUT^1000T/C^/pcDNA3.1(+) and MUT^840delT^/pcDNA3.1(+) was decreased (8.5 ± 2.6% and 6.9 ± 2.4%) (Figures 3(b) and 3(c)). Acridine orange and ethidium bromide staining also suggested that the apoptosis rate was decreased dramatically in cells stably transfected with mutant *Wisp3*; there were no apparent apoptotic cells in them (Figures 3(d)–3(f)).

### 3.3. Dysfunction of Collagen Production in Cells Transfected with Mutants

**Figure 4:**
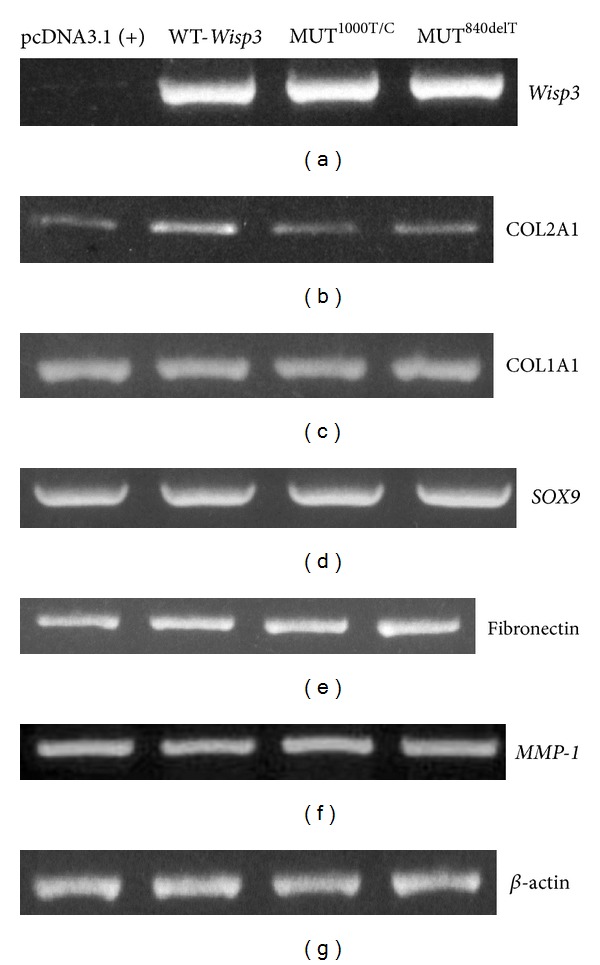
mRNA expression of cartilage-specific genes in C20/A4 cells transfected with wild and mutant *Wisp3*. Cells stably transfected with WT-*Wisp3*/pcDNA3.1(+), MUT^1000T/C^/pcDNA3.1(+), MUT^840delT^/pcDNA3.1(+), or empty vector. mRNA expression of cartilage-specific genes in C20/A4 cells was determined by RT-PCR. (a)–(g) represent *Wisp3*, COL2A1, COL1A1, *SOX9*, fibronectin, *MMP-1*, and *β*-actin separately.

**Figure 5:**
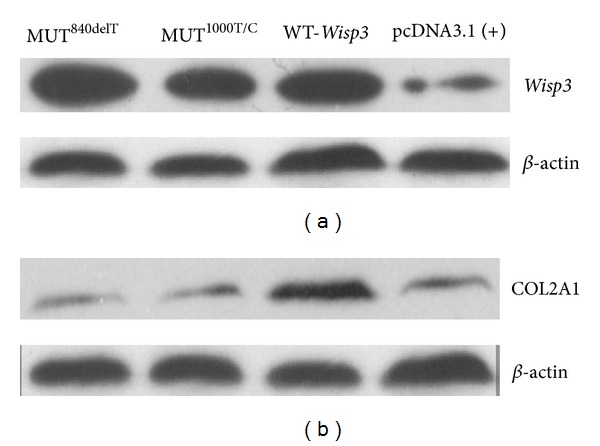
*Wisp3* and COL2A1 protein expression in C20/A4 cells transfected with wild and mutant *Wisp3* analyzed by western blot.

The function of *Wisp3* gene in chondrocytes and the mechanism of disorders in cartilage tissue caused by *Wisp3* mutation were still unclear. The C-20/A4 chondrocytes lines express very low levels of *Wisp3* (Figures 4(a) and 5(a)) and cartilage specific collagens. To investigate the function of wild and mutant *Wisp3* gene in chondrocytes, the mutant and control plasmids were transfected stably into chondrocyte cell line C-20/A4. The WT-*Wisp3*/pcDNA3.1(+) transfected C-20/A4 cells expressed 3.3-fold higher levels of COL2A1 mRNA than the cells transfected with the control vector (Figure 4(b)).  Figure 5(b) demonstrated that stable transfection of C-20/A4 cells with WT-*Wisp3*/pcDNA3.1(+) upregulates type II collagen protein expression (*P* < 0.05). However, the COL2A1 expression did not change in C-20/A4 cells transfected with MUT^1000T/C^ /pcDNA3.1(+) and MUT^840delT^/pcDNA3.1(+) both in mRNA and protein level, compared with the cells transfected with the empty vector.

In contrast, minimal changes were observed in the levels of mRNA of type I collagen, *SOX9*, and fibronectin in response to either wild or mutant *Wisp3* (Figures 4(c), 4(d), and 4(e)). The mRNA expression of *MMP-1*, which had been found dramatically decreased in articular chondrocytes separated from SEDT-PA/PPD patient [15] (Figure 4(f)), wasn't changed in the mutant *Wisp3* transfected chondrocytes. 

### 3.4. Abnormal Intracellular Collagen Content and Secretion in Mutant Chondrocyte

**Figure 6:**
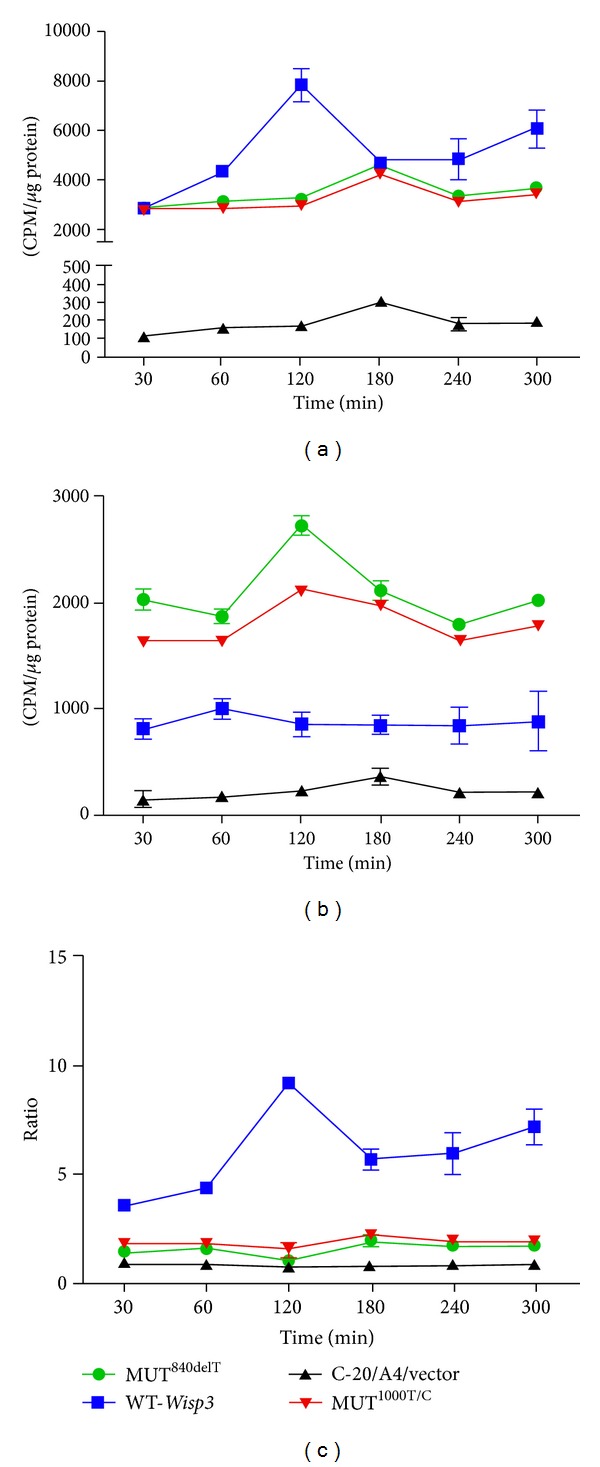
Change of intracellular collagen content and extracellular collagen secretion in mutant chondrocytes analyzed by ^14^C-proline incorporation assay. (a) Time course of ^14^C-proline labeled collagen (detected by radioactivity) secreted to the supernatant of the cultured chondrocytes. (b) Time course of ^14^C-proline labeled collagen content in cultured chondrocytes. (c) Ratio of secreted collagen to intracellular collagen.

By ^14^C-proline incorporation analysis, very low radioactivity was detected in the supernatant of and cell lysate of C-20/A4 cells transfected with control vectors, which indicated that very low collagen synthesis and secretion in this cell line (Figures 6(a) and 6(b)), and the ratio of extracellular collagen secretion to intracellular content is approximately 1. However, in wild type *Wisp3* stably transfected C-20/A4 cells, high radioactivity, were detected in the culture supernatant (3000 CPM to 7000 CPM) and cell lysate (700 CPM to 1000 CPM); the peak collagen secretion and intracellular content were appeared at 120 min and 60 min separately after refreshment of the complete medium, compared to C-20/A4 cells transfected with control vector; *Wisp3* increased the intracellular collagen content to about 5–10 times (*P* < 0.01), and especially increased the extracellular collagen secretion to 10–20 times (*P* < 0.01), and the ratio of extracellular collagen secretion to intracellular content is 3.5–10 (Figure 6(c)). In mutant *Wisp3 *(MUT^840delT^ and MUT^1000T/C^) transfected cells, the peak collagen secretion and intracellular content were backward to 120 min and 180 min separately, although the radioactivity of collagen secretion was slightly higher than that of intracellular collagen content, the extracellular collagen secretion was decreased obviously compared to the wild type *Wisp3* transfected cells, and the ratio of extracellular collagen secretion to intracellular content is about 1.5. 

## 4. Discussion

PPD was attributed to mutations of *Wisp3* gene; we previously identified a novel compound heterozygous mutation (840delT/T1000C) of *Wisp3* in a SEDT-PA/PPD family, and this mutation results in a dramatic decrease in the tensile strength of articular cartilage; however, the detail mechanism is not clear. 

By bioinformatics analysis, we predicted that the compound heterozygous mutation formed a truncated Wisp protein and a Ser334Pro mutated proteins [14]. The 3D-conformational change of the 840delT truncated mutant *Wisp3* protein is the single long peptide loop in the region from signal peptide to the beginning 24 amino acid residues in the first domain (IGFBP) which was subjected to folding into two smaller cross-loops accompanied with a much shorter C-terminus. It has been noted that the function of the first (IGFBP) domain of *Wisp3* is involved in inhibiting the function of IGF-1 to the chondrocytes and the fourth (CK) domain is involved in disulfide-linked dimerisation and is necessary for dimer formation in the endoplasmic reticulum, an important function for the establishment and maintenance of normal 3D-conformation of *Wisp3* protein [18–20]. Through GFP labeled protein localization analysis, we found that wild type *Wisp3* protein did localize in cytoplasm and cell membrane of C-20/A4 cells, but the two mutated *Wisp3* proteins aggregated abnormally in cytoplasm of C-20/A4 cells transfected with MUT^1000T/C^ and MUT^840delT^. It needs further research to validate the hypothesis that 3D-conformational change causes localization change of mutated *Wisp3* protein.

*Wisp3* belongs to the CCN family of proteins, which play important roles in development during chondrogenesis and enchondromatosis and encode cysteine-rich secreted proteins with roles in cell growth and differentiation [21]. To investigate the effects of the T1000C and 840delT mutations on *Wisp3* function in chondrocytes, we compared biological behaviors in C-20/A4 cells transfected separately with WT-*Wisp3*, MUT^1000T/C^, and MUT^840delT^. MUT^1000T/C^ *Wisp3* and MUT^840delT^ *Wisp3* increased proliferation activity as well as decreased apoptosis of C-20/A4 cells obviously, which shared the phenotype of articular chondrocytes (ACs), separated from SEDT-PA patients we described before [15]. Therefore, inhibition of cell proliferation and promotion of precursor cell differentiation are major effects of *Wisp3* on chondrocytes, through which *Wisp3* modulates the balance of cartilage metabolism.

We previously found that PPD cartilage had lost its flexibility, and the main matrix component of cartilage is collagen, so we detected the effect of *Wisp3* gene mutation on the collagen expression in chondrocytes. The results demonstrated that both mutant *Wisp3* lose the function to modulate the expression of cartilage specific matrix type II collagen when compared with wild *Wisp3*, which consisted of results in C-28/I2 and T/C-28a2 cells transfected with another SEDT-PA related *Wisp3* mutation (Cis78-Arg) [22], but the modulation effect of *Wisp3* may not be via activation of *SOX9* in our study since no change of SOX expression was found. After the collagen synthesis, it need to be secreted into the extracellular matrix, if the collagen secretion was changed by gene mutation or 3D-conformational alteration, the function of cartilage will be abnormal, and to further study the dynamic collagen synthesis and secretion, we use ^14^C-proline, which is the major material of collagen synthesis and a major determinant of collagen tertiary structure, to label the new synthesized collagen, through detection of the radioactivity in cell lysate and supernatant to quantify the intracellular collagen content and extracellular secretion at different time points. Compared to the wild type *Wisp3*, MUT^1000T/C^ or MUT^840delT^ *Wisp3* lost the function of increasing the extracellular collagen secretion, delaying the intracellular collagen synthesis, which is one of the important mechanisms for the collage size and density decrease in PPD cartilage described previously. 

 However, we could not find the difference of *MMP-1* mRNA levels between the C-20/A4 cells transfected with wild and mutant *Wisp3*, which was dramatically decreased in ACs separated from SEDT-PA/PPD patients compared with normal ACs. The paradoxical phenomenon may be related to the following causes: (1) C-20/A4 is an immortalized cell line derived from human juvenile costal cartilage and is highly proliferative and not contact-inhibited compared with primary cells, which may have influence on the expressions of matrix and other genes at reasonable levels [23]. (2) MUT^1000T/C^ or MUT^840delT^ results in obviously changed biological behaviors of chondrocytes; however, that cannot fully represent the effect of compound mutation (840delT/T1000C) on the cartilage metabolism. (3) The low expression level of *Wisp3* in C-20/A4 cell line may have interacted influence on the expressions of other genes. 

## 5. Conclusions

*Wisp3* mutations resulted in abnormal protein distribution and dysfunction of cell proliferation, collagen production, and dynamic secretion in chondrocytes, which may be involved in the pathogenesis of SEDT-PA.

## Figures and Tables

**Table 1:** Primer pairs and experimental conditions.

Gene product	Forward and reverse primers (5′-3′)	Expected product size, bp	Annealing temperature (°C)
COL2A1	CCTAATGGAGATGCTGGTCG	187 bp	57
	CCAGGGAATCCAATGTTGC		
COL1A1	ATCCAGCTGACCTTCCTGCG	322 bp	60
	TCGAAGCCGAATTCCTGGTCT		
*Wisp3*	GTAAGCTTAGCGACATGCAGGGGCTCCTCTT	1065 bp	62
	GCGGATCCTTACAGAATCTTGAGCTCAG		
Fibronectin	GTGTGACCCTCATGAGGCAAC	299 bp	60
	TACTCTCGGGAATCTTCTCTGT		
*SOX9*	CACACTACAGCCCCTCCTAC	258 bp	60
	CCTCCTCAAGGTCGAGTGAG		
*MMP-1*	ATGCTGAAACCCTGAAGGTG	305 bp	60
	CAAGATTTCCTCCAGGTCCA		
*β*-actin	TCCTGTGCATCCACGAAACT	310 bp	58
	GAAGCATTTGCGGTGGACGAT		
